# Epidural Catheter Migration in a Patient with Severe Spinal Stenosis

**DOI:** 10.1155/2016/6124086

**Published:** 2016-12-20

**Authors:** Daryl I. Smith, Ryan Anderson

**Affiliations:** Acute Pain Service, Department of Anesthesiology, University of Rochester School of Medicine and Dentistry, 601 Elmwood Ave, P.O. Box 604, Rochester, NY 14642, USA

## Abstract

Establishment of appropriate neuraxial catheter positioning is typically a straightforward procedural undertaking. It can, however, lead to deception of even the most experienced clinician and occur despite the most meticulous attention to detail. Written and verbal consent were obtained from the patient to prepare, discuss, and publish this case report; we describe the occurrence of what we believe was the intraoperative migration of an epidural catheter in the setting of significant tissue changes resulting from a previous spinal fusion.

## 1. Introduction

Lumbar epidurals have been used for control of postoperative pain for a number of years [1]. They can be beneficial in decreasing the total amount of perioperative narcotics consumed as well as improving patient satisfaction scores for pain control. In the presence of certain conditions such as spinal stenosis, previous lumbar disc surgeries, and history of back trauma, epidural catheter placement can be difficult and pain control may be less than adequate. If the catheter is placed in the subdural space or within the thecal sac, dangerous outcomes may arise when clinically relevant epidural doses of medications are subsequently administered [2].

## 2. Case Report

An elective right proximal femur replacement was scheduled for a 70-year-old, 79 kg female. The patient had a past medical history which included severe lumbar stenosis, previous lumber spine fusion from L3 to L5, severe arthritis, coronary artery disease, scleroderma, asthma, and pulmonary hypertension. A lumbar epidural was placed at the T12-L1 level in the operating suite after two attempts and prior to the induction of general anesthesia. A closed tip, multiorifice catheter was used. The block was intended to provide postoperative analgesia. The loss-of-resistance to saline technique was used and the procedure was performed with the patient in the sitting position. No cerebrospinal fluid was noted upon aspiration via the catheter and a 3 mL test dose of 1.5% lidocaine with 1 : 200,000 epinephrine was negative for any hemodynamic or neurodynamic changes. No evidence of an intrathecal catheter placement was present. The catheter was secured and induction of general anesthesia and endotracheal intubation were accomplished uneventfully. The patient received 3.8 liters of crystalloid during the four-hour case. Urine output was 0.5 liters and the estimated blood loss was 0.9 liters. One hour prior to the end of the case, an epidural infusion of 0.0625% bupivacaine with 12 mcg/mL hydromorphone was begun at a rate of 6 mL/hr. This concentration of bupivacaine (which is the lowest of the three preparations available in our pharmacy) was chosen because of lability in the patient's intraoperative blood pressure. No intraoperative epidural bolus dose of local anesthetic was given for the same reason.

In the postanesthesia care unit (PACU), the patient was alert and oriented and reported a pain score of 0/10 on the visual analogue scale (VAS). A cold temperature test yielded a T4 to S1 sensory level bilaterally. A brief episode of hypotension with systolic pressures in the 90s was treated with crystalloid fluid boluses. The patient was subsequently discharged to the ward.

Approximately six hours after arrival, the patient complained of bilateral upper extremity weakness and tongue numbness. Physical examination by the nursing service revealed 5+/5+ hand grip strength, but the patient's speech was noted to be slurred. The acute pain service was called to assess the patient. The patient had minimal lower extremity movement with 1/5 strength. During elevation of the head of her bed from a 15 degree recline to a 45 degree recline, she became nauseated and vomited. The sensory level was unchanged from the postoperative assessment and the epidural catheter depth was found to be unchanged. A 1 mL bolus of 1.5% lidocaine with 1 : 200,000 epinephrine was given via the epidural catheter. This resulted in no increase in heart rate or blood pressure, but the patient immediately reported “prickling” sensations in her arms. Based upon her previous episode of systolic hypotension and sensory loss greater than expected from the dose of local anesthetic previously administered, a hydromorphone epidural infusion without local anesthetic was begun. This combination was chosen because the hydromorphone alone could provide some degree of analgesia at a safe concentration for both the epidural space and any possible intrathecal deposition.

Three hours later, the patient reported increased sensation and strength in both lower extremities. This allowed her to participate in physical and occupational therapy. She continued to report unchanged lingual numbness. The epidural solution was stopped and oral hydrocodone-acetaminophen was begun.

**Figure 1:**
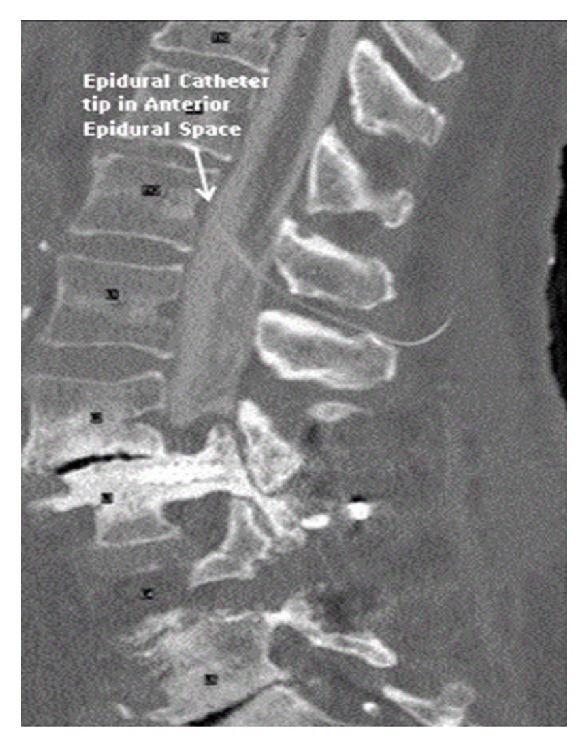
Epidural catheter tip positioned in the anterior epidural space with the catheter traversing the thecal sac. Note contrast only in the intrathecal compartment.

On the second postoperative day, she reported that her leg weakness and lingual paresthesias had disappeared. A CT myelogram through the epidural catheter was performed and the catheter was noted to enter and traverse the thecal sac with contrast material found only in the intrathecal compartment. The tip of the epidural catheter tip was identified in the anterior epidural space (Figure 1).

**Figure 2:**
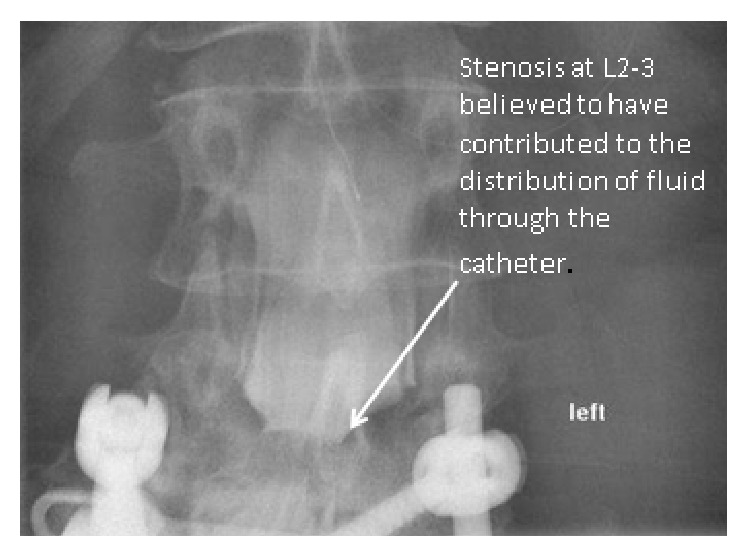
Severe central canal stenosis at L2-3 which is believed to have contributed to the distribution of fluid injected through the catheter.

Severe spinal stenosis was noted at the L2-L3 vertebral level and was considered by the radiologist to have contributed to the distribution of contrast material injected through the catheter (Figure 2).

**Figure 3:**
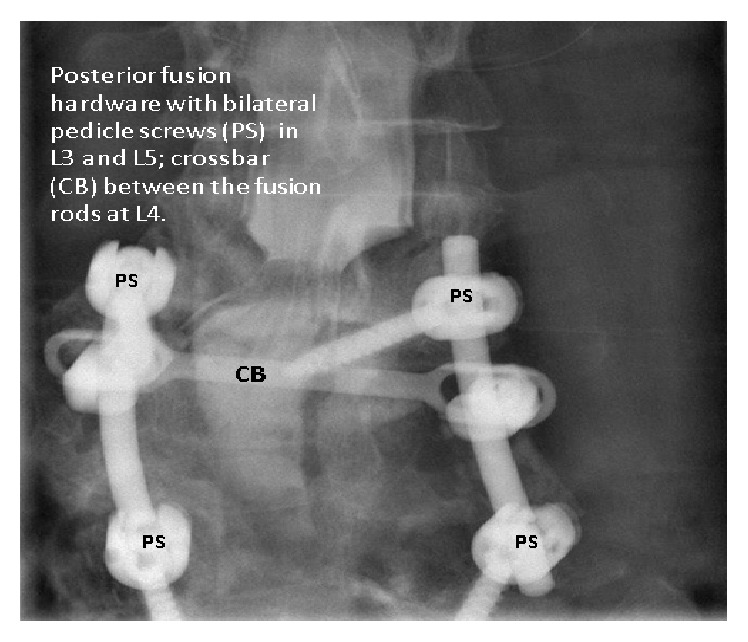
Surgical changes include posterior fusion hardware with bilateral pedicle screws in L3 and L5; crossbar between the fusion rods at L4.

In addition, left lateral listhesis of L2 on L3; grade 1 anterolisthesis of L4 and L5; and surgical changes which included posterior fusion hardware with bilateral pedicle screws in L3 and L5 were also seen. There was no radiographic evidence of hardware failure (Figure 3).

The catheter was removed following the CT study with the tip intact. No malformation of the catheter was noted. The patient was discharged to a skilled nursing facility for rehabilitation on the sixth postoperative day and has had an uneventful recovery as of her 1 year follow-up visit with the orthopedic surgical service.

## 3. Discussion

Subdural placement of an epidural catheter has a highly variable clinical presentation [1]. The reported incidence of unintentional subdural placement of epidural catheters is as high as 7–11% [1]. The subdural space is a potential space. It arises from iatrogenic or pathologic dissection of thecal membranes from the epidural space. A natural cleavage plane between dura and arachnoid mater was noted during cadaveric dissections which contained sparse cellular and noncellular connective tissues [1]. It must be noted, in comparison, that the contents of the epidural space are highly heterogeneous. It is therefore understandable that the anesthetic spread and its subsequent effect can vary causing confusion in differentiating between aberrant epidural block characteristics and a subdural local anesthetic injection. Subjects also show great natural variability in fundamental anatomic features of the spinal canal. Even with normally functioning epidural anesthesia, there are highly varied patterns of solution spread within the epidural space [3]. Patients with unexpected extent of neuromuscular blockade, duration, intensity, or hemodynamics may be manifesting both subdural and epidural distribution of local anesthetics [4].

The “classic” subdural block, as described in original case reports, presents with several key features. These include excessive sensory blockade, sympatholysis out of proportion to local anesthetic dose, variable motor blockade, respiratory distress, unconsciousness, and even cardiac arrest [1].

Several risk factors increase the chances of subdural placement of an intended epidural catheter. These include catheterization following lumbar puncture usually discovered via a positive test dose or CSF aspiration; catheterization following previous subdural injection; epidural needle rotation before catheter insertion; and technically difficult block placement. The unifying theme behind the risk factors is damage to the dura mater [1].

The dura mater is too thin to be resolved by imaging in most cases [4], and the location of the arachnoid membrane can only be inferred based upon the pattern of contrast distribution. These patterns include a smooth layering of contrast against the inside of the dural sac; and a lack of solution passage into the intervertebral foramina [4]. Radiologic visualization of the neuraxial catheter may be helpful when clinical symptoms make the situation difficult to assess [2].

The patient in this case suffered from significant preexisting lumbar disease and had undergone previous lumbar spine surgery. Either may have caused epidural fibrosis. A compact epidural fibrosis can cause compression or stretching of neuromeningeal structures due to restricted mobility. This can lead to arachnoepiduritis with reduced energy substrate delivery to the nerve roots, subsequent tissue edema, and creation of a real subdural space. The lack of prospective data collection and an ultimate clinical standard leave the definitive clinical identification of subdural injection uncertain [4].

## 4. Conclusion

Despite current, frequently used methods of testing appropriate placement of catheters in the neuraxis, there is still a lack of an absolutely reliable means of confirming the location of the catheter in the epidural space. Radiographic examination of radiopaque dye injections is time and resource consuming and not commonly available in most perioperative settings. Variations in the anatomy of the epidural space (even in the absence of pathology or postoperative change) exist in otherwise “normal” individuals. In this case the potential for catheter dislodgement or misplacement was compounded by the temporal and situational component of the procedure. Instead of the catheter being placed in the dedicated block area, the block was performed in the operating room immediately prior to induction of general endotracheal anesthesia for a 4-hour procedure. This allowed time enough for a negative test dose to be confirmed, but not enough time or opportunity for any developing aberrancy in catheter performance to be seen in evolution. Thus a regional anesthesia “perfect storm” was created.

This case is unique in that an ideal setting (preexisting surgical trauma with concomitant, severe postoperative anatomical changes) for such an event to occur was created. It serves as a cautionary tale for trainees and senior practitioners alike. Strict adherence to the principles and practice of neuraxial anesthesia are mandatory for patient safety and clinically acceptable outcomes in ideal situations. When complex preexisting anatomical variants are present, further vigilance, patience, and continuous evaluation must be the standard. This case provides an important example of the value of adequate, preoperative time to place and to fully assess the appropriate function of an indwelling neuraxial catheter beyond the usual negative test dose.

## References

[1] Hoftman N. (2011). Unintentional subdural injection: a complication of neuraxial anesthesia/analgesia. *Anesthesiology Clinics*.

[2] Shin S., Cho Y. Y., Park S. J., Koo B.-N. (2013). Apnea and unconsciousness after accidental subdural placement of an epidural catheter. *Korean Journal of Anesthesiology*.

[3] Hogan Q. (2002). Distribution of solution in the epidural space: examination by cryomicrotome section. *Regional Anesthesia and Pain Medicine*.

[4] Hogan Q. H., Mark L. (2009). Subdural injection: what's the gold standard?. *Regional Anesthesia and Pain Medicine*.

